# Successful surgical management of unresectable stage IIIB LSCC following neoadjuvant chemotherapy combined with toripalimab: a case report

**DOI:** 10.3389/fimmu.2025.1651208

**Published:** 2025-09-08

**Authors:** Qiang Guo, Dan Li, Qiang Liu, Qiao Li, Hua Liu, Tao Liu, Tao Zeng, Xiao-Fei Ren, Jun Zhang, Min Zeng, Hui Liu, Jia-Long Guo

**Affiliations:** ^1^ Department of Cardiothoracic Surgery, Taihe Hospital, Hubei University of Medicine, Shiyan, Hubei, China; ^2^ Department of Oncology, Taihe Hospital, Hubei University of Medicine, Shiyan, Hubei, China; ^3^ Department of Thoracic Surgery, Affiliated Hospital of Zunyi Medical University, Zunyi, Guizhou, China; ^4^ Department of Ultrasound Medicine, Taihe Hospital, Hubei University of Medicine, Shiyan, China

**Keywords:** lung squamous cell carcinoma, chest CT, lymph node metastasis, toripalimab, chemotherapy

## Abstract

Immunotherapy has emerged as a promising strategy to improve the survival of patients with cancer. This case report presents a patient with unresectable advanced lung squamous cell carcinoma (LSCC) who successfully underwent surgical resection following neoadjuvant treatment with toripalimab and chemotherapy. Chest computed tomographic (CT) scan revealed mass lesions in both upper lungs, with the left upper lesion measuring approximately 5.6 × 4.6 cm. A CT-guided biopsy confirmed LSCC in the left upper lung. Enhanced chest CT at our hospital indicated a left upper lobe tumor measuring approximately 5.6 × 5.0 cm, with possible invasion of the left pulmonary artery and multiple enlarged mediastinal and ipsilateral hilar lymph nodes. A nodule in the apical segment of the right upper lobe was noted. The patient was staged as cT4N2M0, stage IIIB, and received three cycles of toripalimab (240 mg) combined with cisplatin (120 mg) and paclitaxel (300 mg). Mild nausea occurred during treatment. Follow-up enhanced CT demonstrated the left upper lobe lesion reduced to approximately 4.4 × 3.3 cm. The patient subsequently underwent thoracoscopic left upper lobectomy, mediastinal lymphadenectomy, and pulmonary artery reconstruction after treatment. Postoperative symptomatic supportive care included infection control, airway clearance, and nebulization. The patient was discharged on the sixth postoperative day. This case demonstrates that immunochemotherapy may enable surgical intervention in patients with unresectable lung cancer, potentially improving patient outcomes and informing future clinical practice.

## Background

**Figure 1:**
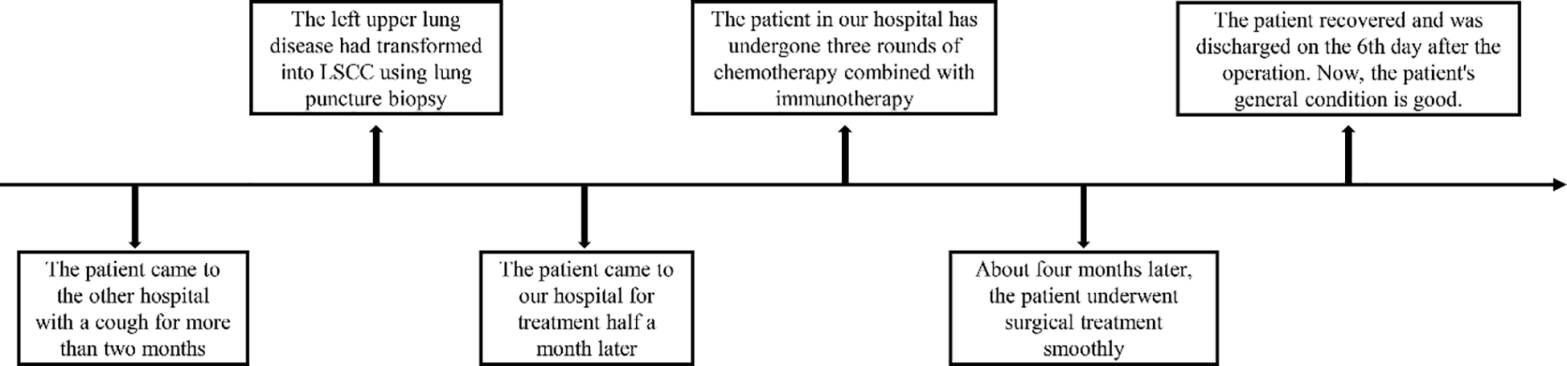
Flowchart of the patient’s treatment process.

Lung squamous cell carcinoma (LSCC) is one of the most prevalent malignant lung tumors. While early-stage LSCC can be effectively treated with surgery, advanced stages frequently have poor outcomes due to inoperability. Current research indicates that neoadjuvant therapy can induce tumor shrinkage in some patients with locally advanced LSCC, making them eligible for surgery. This case report describes a patient whose chest CT scan revealed mass lesions in both upper lungs, including a left upper lung lesion measuring approximately 5.6 × 4.6 cm. Following three cycles of toripalimab in combination with chemotherapy, the tumor burden was significantly reduced, allowing for surgical resection (**Figure 1**). This case highlights the potential of immunochemotherapy to convert unresectable lung cancer into a surgically treatable condition, offering a promising strategy to extend survival and guide clinical decision-making.

## Case report

A 74-year-old female patient presented with a persistent cough lasting over two months and underwent a chest CT at the People’s Hospital of Shiyan City, Hubei Province. The scan revealed a mass in the left upper lung measuring approximately 56 × 46 mm, highly suspicious for malignancy. A smaller-sized lesion was also identified in the right upper lung, suggestive of an early-stage tumor. Bronchoscopy showed no significant abnormalities. Given the bilateral lesions, a primary lung tumor was considered. Due to the prominent mass in the left upper lung, a CT-guided biopsy was performed, and postoperative pathology confirmed squamous cell carcinoma (SCC) in the left upper lobe.

**Figure 2:**
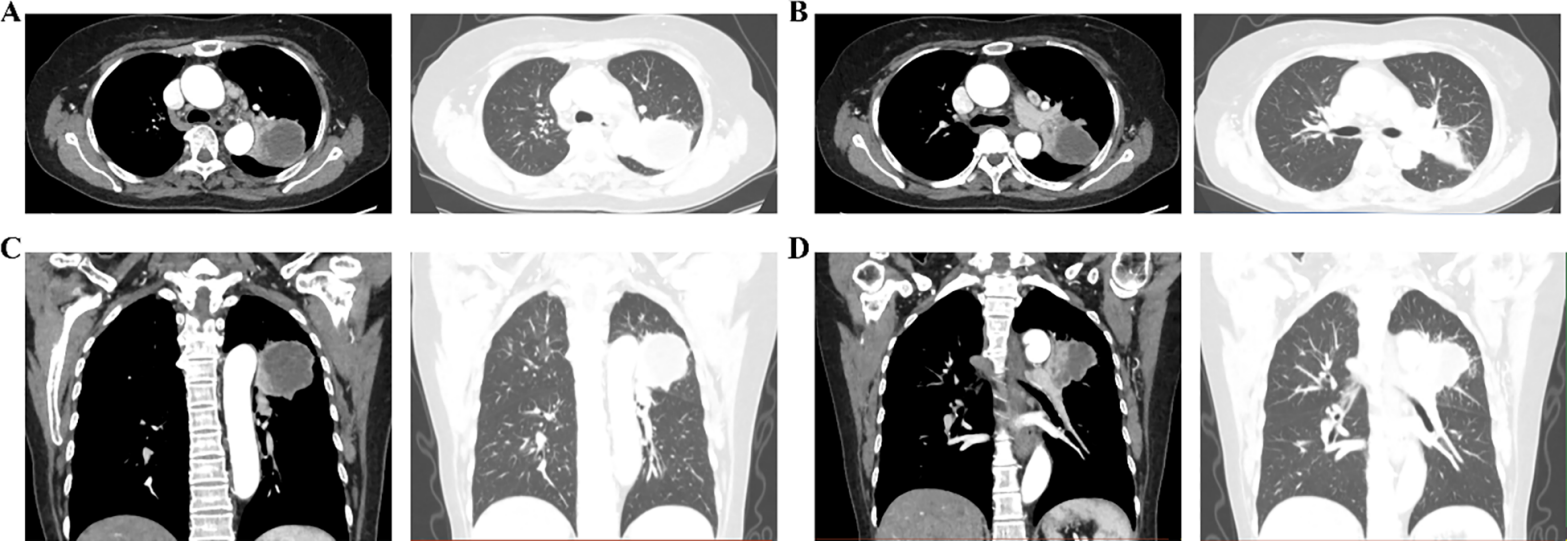
Enhanced chest CT showing the upper lobe lesion in the left lung. **(A, B)** Mediastinal and lung windows at different levels; **(C, D)** Three-dimensional reconstruction of lesion distribution.

**Figure 3:**
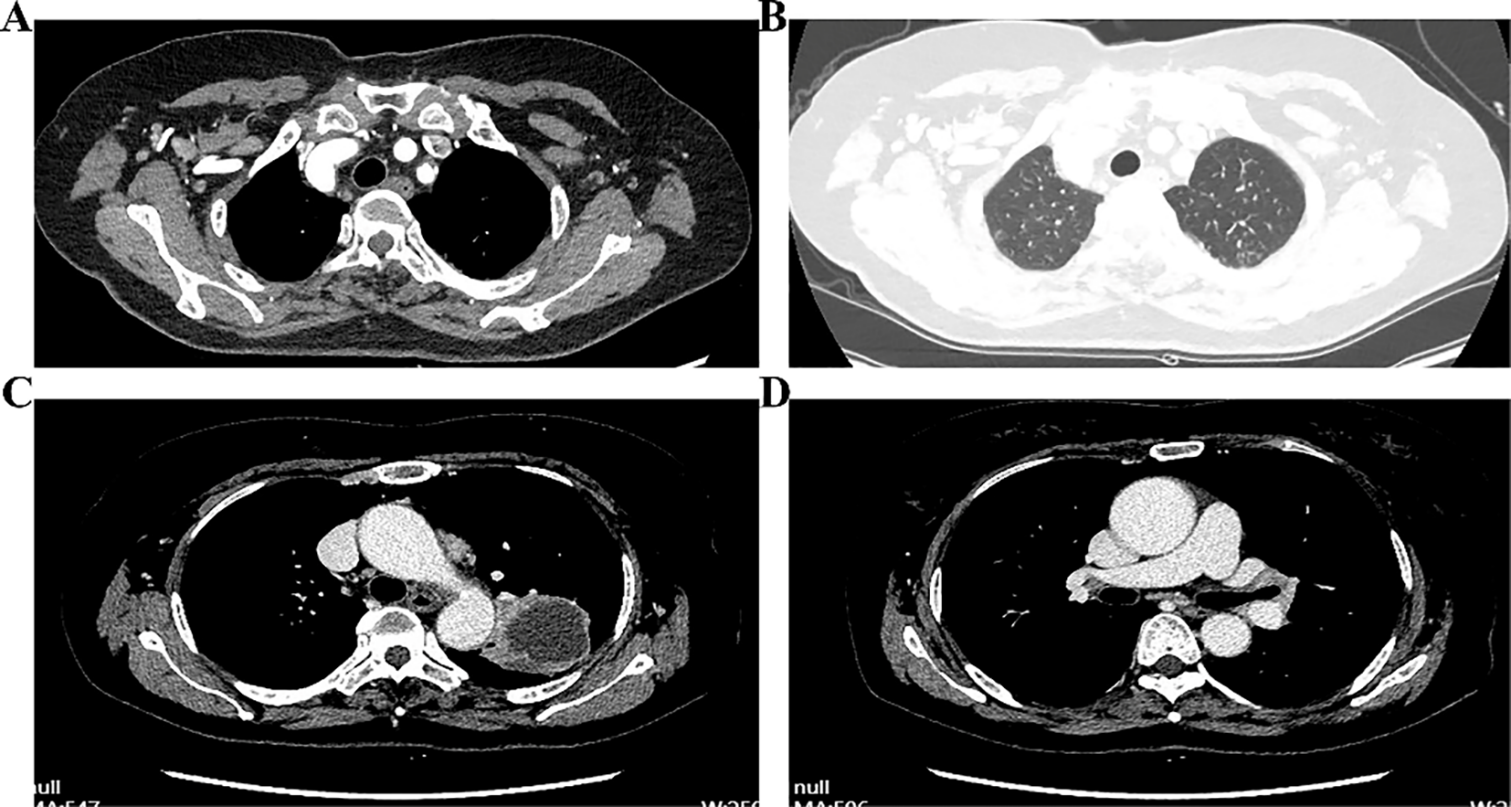
Enhanced chest CT showing the right upper lung lesion. **(A, B)** Ground-glass nodules in the upper right lung using lung window and mediastinal window; **(C, D)** Mediastinal lymph nodes using mediastinal window.

Enhanced chest CT at our hospital further confirmed LSCC in the left upper lobe, with a lesion measuring approximately 5.6 × 5.0 cm, potential invasion of the left pulmonary artery, enlarged lymph nodes in the mediastinum and left hilum, raising concern for metastasis, and a nodule of an approximate diameter of 0.8 cm was observed in the apical segment of the right upper lobe (**Figures 2A-D**, **3A-D**). Venous ultrasound of the lower limbs showed bilateral thrombosis in the calf muscle veins. Cardiac ultrasound revealed left atrial enlargement, degenerative and calcific changes of the aortic valve with moderate to severe regurgitation, and an ejection fraction of 56%. Positron emission tomography–computed tomography (PET/CT) demonstrated increased metabolic activity in the posterior segment of the left upper lobe mass, supporting malignancy. Hypermetabolism was also observed in multiple lymph nodes in the left hilum and mediastinum, suggesting metastatic spread. Based on the imaging and clinical findings, the patient was staged as cT4N2M0 (stage IIIB). Direct surgical intervention was deemed high risk due to probable involvement of the left pulmonary artery and the complexity of a radical resection. Following multidisciplinary discussion by our hospital’s lung cancer multidisciplinary team (MDT), preoperative neoadjuvant therapy was recommended. After a detailed discussion of the treatment plan and associated risks, the patient and her family consented to a regimen comprising chemotherapy (cisplatin: 120 mg; paclitaxel: 300 mg) combined with immunotherapy (toripalimab: 240 mg). The patient experienced mild nausea during treatment but reported no other significant adverse effects.

**Figure 4:**
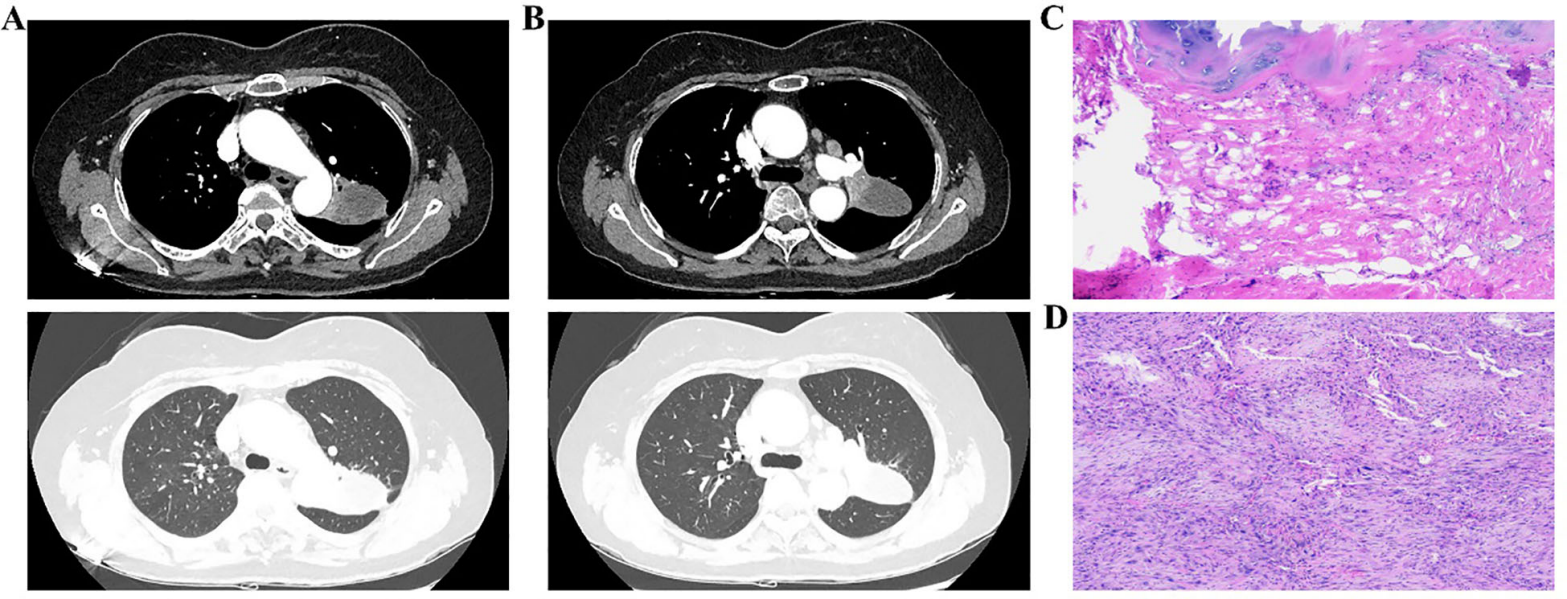
Enhanced chest CT, intraoperative bronchial margin assessment, and postoperative pathology of left upper lung lesions. **(A, B)** Post-treatment CT images of different facets; **(C)** Rapid intraoperative pathology of the bronchial margin; **(D)** Postoperative pathology of the left upper lobe lesion.

Approximately four months after the diagnosis of left upper lobe LSCC, enhanced chest CT revealed that the tumor had grown to approximately 4.4 × 3.3 cm, with multiple lymph node metastases in the mediastinum and left hilum, along with possible invasion of the left pulmonary artery. The nodule in the apical segment of the right upper lung persisted, with potential malignancy pending further evaluation (**Figures 4A, B**). Pulmonary bullae were again noted in the left lower lung. Bronchoscopy revealed inflammatory changes in the bronchus, with no noticeable tumor lesions. After the patient and her family were informed of the surgical plan and associated risks, both provided consent, and the operation was scheduled for April 22, 2025.

Under general anesthesia, the patient was positioned in the right lateral decubitus position, followed by routine disinfection and draping. A 1.0 cm incision was made along the left seventh intercostal space at the mid-axillary line to serve as the observation port. Two additional incisions–approximately 3.0 cm at the left fourth intercostal space along the anterior axillary line, and 2.0 cm at the left eighth intercostal space along the posterior axillary line–were created as operational ports. Thoracoscopic examination revealed adhesions, which were lysed. The lesion appeared encased near the hilum of the left upper lobe. The lower pulmonary ligament and mediastinal pleura were dissected. Multiple lymph nodes were excised, including paratracheal, subaortic, paraaortic, and subcarinal. The main trunk of the left upper pulmonary artery was isolated and double-ligated with 7 – 0 sutures for future control. A tunnel was created at the midpoint of the oblique fissure by opening the mediastinal pleura, and the underdeveloped anterior oblique fissure was resected using a linear cutter (Johnson & Johnson, USA). A posterior oblique fissure tunnel was established. Due to suspected lower lobe involvement, part of the lower lobe tissue was resected with the linear cutter. The left upper pulmonary vein was dissected, closed, and divided using the linear cutter. Interlobar lymph nodes were cleared, and the lingular artery was fully exposed and similarly transected. The lower pulmonary artery was carefully mobilized. Although efforts were made to dissect the apical segment artery, tumor invasion was evident, necessitating temporary occlusion of both the main pulmonary artery and the left lower lobe artery. The apical segment artery was transected with scissors and sealed using a vascular stapler (Johnson & Johnson, USA). The anterior segment artery was exposed and partially resected with a linear cutter due to tumor infiltration, resulting in the resection of the left upper lobe. Although no arterial stenosis was observed, minor bleeding was noted at the resection margin. The specimen was removed through the fourth intercostal space. Intraoperative frozen section analysis of the bronchial stump confirmed the absence of tumor cells (**Figure 4C**). The thoracic cavity was irrigated, and the lung was inflated to check for leaks. No air leakage was detected at the bronchial stump or the remaining left lower lobe. Hemostasis was thoroughly achieved. The surgical site was packed with hemostatic gauze (Johnson & Johnson, USA). The bronchial stump and vascular margins were covered with bio-adhesive. A chest tube was placed in the left seventh intercostal space. Following confirmation of instrument count, routine closure was performed.

**Figure 5:**
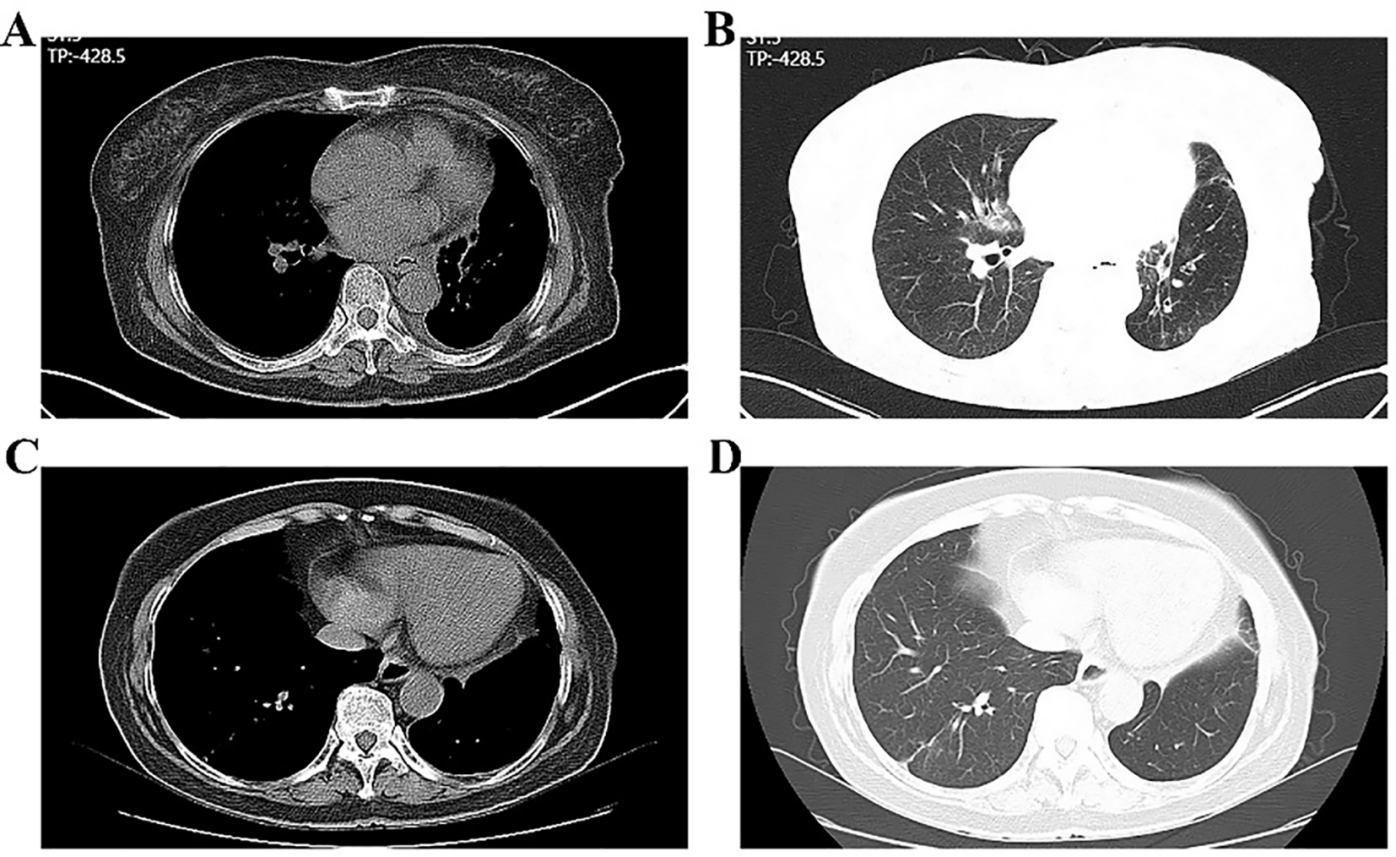
Postoperative plain chest CT of the patient. **(A, B)** Before discharge; **(C, D)** One month after discharge.

Postoperative pathology staged the disease as ypT2bN2M0, stage IIIA (**Figure 4D**). Histopathology confirmed SCC in the left upper lobe, measuring 4.8 cm × 4.6 cm × 3.5 cm, with areas of necrosis, pleural invasion, and bronchial infiltration, but no vascular tumor or perineural invasion. Treatment response showed 50% viable tumor cells, 20% necrosis, and 30% stromal component. No tumor metastasis was found in three peribronchial lymph nodes. One of the three subaortic lymph nodes exhibited tumor metastasis (1/3). No metastasis was detected in the lymph nodes from the subcarinal (0/3), hilar (0/1), lower pulmonary ligament (0/2), paratracheal (0/3), paraaortic (0/4), or interlobar (0/3) regions. A chest CT scan performed on postoperative day 6 indicated changes consistent with left upper lobe carcinoma resection. A persistent nodule in the right upper lung apex was noted, warranting further evaluation. Pulmonary bullae were again observed in the left lower lobe (**Figures 5A, B**). The patient was discharged in stable condition on postoperative day six. One month later, a follow-up chest CT revealed post-surgical changes in the left upper lobe and improved infection status in the right middle and left lower lobes. The ground-glass nodule in the apical segment of the right upper lung persisted and required further evaluation. Pulmonary bullae in the left lower lung were also reidentified (**Figures 5C, D**).

## Discussion

LSCC is a common subtype of lung cancer strongly associated with smoking and typically arises in the main bronchi. Early disease is frequently asymptomatic or presents as a persistent dry cough. Diagnosis is usually established by chest CT scans followed by bronchoscopy and biopsy. In recent years, particularly following the coronavirus disease 2019 (COVID-19) pandemic, incidental pulmonary mass lesions have been frequently detected on chest CT scans (1–3). However, some patients did not undergo routine screening during that period. In this case, the patient presented with chronic cough, and chest CT revealed a 5.5-cm mass in the left upper lung, with possible invasion of the left pulmonary artery and enlarged mediastinal and hilar lymph nodes, consistent with cT4N2M0, stage IIIB disease. At diagnosis, the patient had locally advanced disease and was initially considered unsuitable for surgical treatment, reflecting the advanced cancer stage and poor prognosis.

Patients with early-stage LSCC often benefit from surgery, but for those with locally advanced disease, surgical options remain limited, and the prognosis is generally poor. In our case, the left upper LSCC was locally advanced, and a ground-glass nodule in the right upper lung apex was suspected to be malignant, though the primary determinant of survival was the left-sided lesion; thus, the right lung nodule required no immediate intervention. Recent studies support neoadjuvant chemotherapy combined with immunotherapy for patients with resectable locally advanced LSCC to downstage tumors and create opportunities for curative resection (4, 5). In this case, three cycles of preoperative chemotherapy combined with immunotherapy significantly reduced the left upper lobe lesion, enabling surgical resection. Our report provides a more detailed account of the treatment process and surgical complexity (including the need for pulmonary artery occlusion) than most published studies. Moreover, clinical reports on the efficacy of the Chinese-produced monoclonal antibody (toripalimab) remain limited, making these findings particularly noteworthy.

Surgical resection following neoadjuvant therapy proved technically demanding. Dense thoracic adhesions were encountered intraoperatively, and the left upper lung lesion near the hilum appeared encased. This procedure required dissection and control of the main trunk of the left upper pulmonary artery, which was double-ligated for vascular safety. Possible lower lobe involvement necessitated partial removal of the dorsal segment using a linear cutter during resection. Subsequently, after mobilizing the lower lobe artery, the apical segment artery was attempted to be dissected. However, tumor invasion necessitated temporary occlusion of both the main pulmonary artery and the origin of the lower lobe artery. The apical segment artery was transected with scissors and closed using a vascular stapler. The anterior segment artery was exposed, and due to tumor involvement, a portion of its wall was resected with a linear cutter. Despite these technical challenges, the patient successfully underwent minimally invasive surgery, which is commendable for a third-tier city. Following this advanced therapeutic approach, the patient was discharged in good condition on postoperative day 6.

In today’s rapidly evolving era, the diagnostic value of chest CT in lung cancer is indisputable. Treatment must keep pace with current advancements, and third-tier cities must integrate modern strategies to improve outcomes. Whether surgical intervention is warranted for the ground-glass nodule in the right upper lung remains under consideration. Overall, this case illustrates that a patient initially considered inoperable successfully underwent surgical treatment following combined chemotherapy and immunotherapy, highlighting the potential to extend survival and informing future treatment strategies.

## Data Availability

The original contributions presented in the study are included in the article/supplementary material. Further inquiries can be directed to the corresponding authors.
